# Clinical effects, mechanisms and spread of erector spinae plane block and paravertebral block in thoracic and breast surgery: a narrative review

**DOI:** 10.1097/JS9.0000000000003135

**Published:** 2025-08-05

**Authors:** You Chen Zhang, Ye Sun, Shu Han Li, Shi Jie Ma, Xi Yue Wu, Jing Ya Gao, Xiang Zheng Qin

**Affiliations:** a Department of Clinical Medicine, Medical College of Yanbian University, Yanji, Jilin Province, China; b Department of Anatomy, Medical College of Yanbian University, Yanji, Jilin Province, China

**Keywords:** analgesia, anatomical mechanism, erector spinae plane block (ESPB), extent of spread, paravertebral block (PVB), thoracic surgery

## Abstract

Resolving the controversies surrounding the anatomical spread, clinical effectiveness and safety of erector spinae plane block (ESPB) versus paravertebral block (PVB) is crucial for optimizing postoperative pain management in thoracic and breast surgery. This review systematically examines evidence published between 1 January 2014 and 1 January 2025, regarding the clinical efficacy, anatomical mechanisms, and complication profiles of ESPB and PVB, with a specific focus on their application in video-assisted thoracoscopic and breast surgery. Both ESPB and PVB significantly reduce postoperative pain and opioid consumption compared to controls (*P* < 0.05), thereby minimizing opioid-related complications. However, important controversies persist over their comparative analgesic performance and safety. Some studies report that PVB may provide superior analgesia, but it also carries a higher risk of complications, such as hematoma (2 cases in the PVB group, none in the ESPB group) and pneumothorax (observed with PVB but not reported with ESPB). ESPB is therefore considered safer due to its lower complication rate. Anatomical investigations reveal that ESPB exhibits multidirectional spread of local anesthetic: (1) cranio-caudal along the fascial plane, increasing the number of dermatomes covered and expanding the area of analgesia; (2) medial spread to the paravertebral space, which may enhance blockade of the ventral rami and improve analgesic reliability; (3) lateral diffusion toward the intercostal spaces, potentially influencing chest wall sensation; (4) spread to the dorsal rami, contributing to posterior thoracic analgesia; and (5) limited anterior extension, which has implications for the consistency of ventral ramus blockade. The clinical significance of these spread directions lies in their impact on the quality, extent, and predictability of analgesia, as well as the potential for reducing procedure-related risks compared to PVB. Given current uncertainties in anatomy and outcomes, immediate research priorities should include developing individualized ESPB protocols that account for anatomical variation and total local anesthetic dose, and validating these approaches through multicenter randomized trials. By clarifying these issues, this review aims to provide clinicians with focused, up-to-date evidence to guide block selection, optimize perioperative outcomes, and support the standardization of regional anesthesia protocols.

## Introduction

Postoperative pain represents one of the most pressing and pervasive challenges after thoracic and breast surgeries. Epidemiological studies have shown that moderate-to-severe acute pain is experienced by 30–50% of patients following thoracic surgery and by 35–60% of patients after breast surgery^[1,2]^. Moreover, persistent pain becomes chronic in 25–50% of thoracic surgery patients and in 20–50% of breast surgery patients within the first year after surgery^[3-6]^. Unrelieved pain not only compromises patient comfort but can delay recovery, impair respiratory function and mobility, and contribute to the development of chronic postsurgical pain syndromes.

**HIGHLIGHTS**

- The review suggests that the diffusion pathway of local anesthetics in ESPB exhibits considerable variability, casting doubt on its consistent reach to the paravertebral space.
- The review reveals a critical trade-off: while PVB may provide slightly better pain relief, it carries higher risks of complications such as hematoma and pneumothorax, making ESPB a safer alternative.
- The analysis emphasizes the need for more in-depth anatomical studies and individualized approaches – taking into account factors like injection volume and tissue composition – to optimize these regional anesthesia techniques.

Traditionally, opioid analgesics have formed the mainstay of postoperative pain management in these settings. While effective, opioid-based regimens are accompanied by a host of adverse effects – including nausea, vomiting, constipation, respiratory depression, sedation, and a higher risk of dependence or abuse. The limitations and complications associated with opioids underscore the urgent need for effective, targeted, and opioid-sparing analgesic strategies.

Recent advancements in regional anesthesia have highlighted two important techniques: the erector spinae plane block (ESPB) and thoracic paravertebral block (PVB). ESPB, first described in 2016, is a type of fascial plane block involving the injection of a local anesthetic into the plane between two fascia layers to block nerves within that plane or between adjacent tissues^[7]^. In contrast, PVB is a classic trunk block where a local anesthetic is injected into the external opening of the thoracic intervertebral foramen to block spinal nerve roots^[8]^. Both ESPB and PVB are now commonly used in thoracic, abdominal, pelvic, and breast surgeries as effective methods for postoperative analgesia. A growing body of clinical evidence has demonstrated that ESPB and PVB provide significant reductions in postoperative pain (*P* < 0.05) and opioid use (*P* < 0.05)^[9]^, but also promote faster recovery (*P* < 0.05)^[10]^.

However, several important controversies remain, and there is currently no broad consensus on which technique is superior for specific surgical contexts. Specifically:

1. Comparative analgesic efficacy: Studies differ on whether PVB provides superior pain relief and more opioid sparing than ESPB, especially across different surgical types and patient populations.
2. Mechanisms of action: The primary mechanism underlying ESPB remains under debate; some evidence suggests its efficacy is related to spread into the paravertebral space, while others argue it primarily acts as a fascial plane block.
3. Complication profiles: There are conflicting reports regarding complication rates, such as pneumothorax and hematoma, with some studies indicating differing risk profiles for ESPB and PVB.
4. Anesthetic spread: Imaging and cadaveric studies reveal variable anesthetic distribution with both techniques, and the significance of these patterns for clinical outcomes is still unclear.

This review systematically examines studies from the past decade to clarify the comparative effectiveness, safety, and anatomical mechanisms of ESPB and PVB in thoracic and breast surgery. By addressing the above controversies, we aim to provide clinicians with a practical, evidence-based framework for perioperative pain management decisions and suggest directions for future research and practice.

## Materials and methods

### Search strategy

This is a narrative review article. The two authors independently searched the PubMed and Web of Science databases for articles published between 1 January 2014 and 1 January 2025, using the keywords “erector spinae plane block,” “paravertebral block,” “fascial plane block,” “regional anesthesia,” and “pain.” References from reviews and relevant articles were also examined to identify additional eligible studies, with all potential articles being searched independently by both authors. Initially, the title and abstract of each article were reviewed, irrelevant studies were excluded, and the remaining articles underwent a comprehensive analysis. Any discrepancies were discussed within the group until consensus was reached.

### Inclusion criteria and exclusion criteria

**Figure 1.**
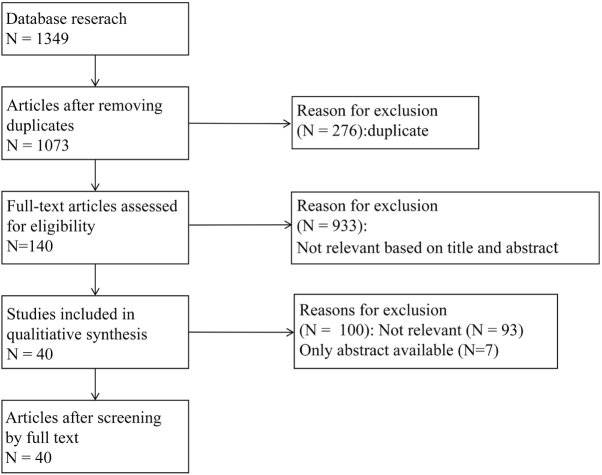
Process of searching and screening literature.

Based on the PICOS framework, studies were included if they: (1) enrolled adult patients who underwent thoracic surgery and breast surgery; (2) patients underwent ESPB or PVB; (3) included a comparator group; (4) reported VAS or NRS pain outcomes; (5) were original research (RCT studies); (6) sample size is more than 60 people. Exclusion criteria: publication type (editorials, letters, reviews, conference abstracts or proceedings, personal communications, or case reports), animal studies, absence of VAS or NRS outcomes, sample size is less than 60 people, and irrelevance to the VATS or breast surgery (based on title and abstract). To mitigate judgment bias, two reviewers independently screened all records. Disagreements were resolved through discussion or, if needed, a third reviewer decided. A total of 1349 articles were retrieved in the preliminary search. After removing duplicates, 1073 articles remained. After reviewing titles and abstracts, 933 articles were excluded based on the inclusion and exclusion criteria, leaving 140 articles. After a thorough review of the full articles, 100 were excluded based on the criteria, and 40 articles were ultimately included (Fig. 1).

### Quality assessment

Two authors evaluated the methodological quality of the trials according to the Cochrane risk-of-bias tool^[11]^. Each item was categorized as having a “low,” “unclear,” or “high” risk of bias. Any uncertainty arose were resolve by discussion between two researches until a consensus was achieved.

### Result

Forty articles were ultimately included in this review, including: 14 anatomical studies and 26 randomized controlled trial (Fig. 1).

### Use of artificial intelligence

No artificial intelligence (AI) tools were used in the conduct of this research or in the preparation of this manuscript. This review is compliant with the TITAN Guidelines 2025 governing the declaration and use of artificial intelligence in scientific research^[12]^. No artificial intelligence (AI) tools were used in the conduct of this research or in the preparation of this manuscript.

## Anatomy

### Erection spinal muscles

The erector spinae muscle group originates from the dorsal surface of the sacrum, the spinous processes of the lumbar vertebrae, the posterior iliac crest, and the lumbar dorsal fascia. The muscle fibers ascend and gradually separate into three longitudinal columns that run parallel from medial to lateral. The lateral column is the iliocostalis muscle (subdivided into lumbar, thoracic, and cervical portions). The intermediate column contains the longissimus muscle (subdivided into thoracic, lumbar, and cervical portions). The medial column forms the spinalis muscle (subdivided into thoracic, cervical, and capitis portions). The erector spinae muscle group inserts into the inferior borders of the ribs, the transverse processes of the cervical and thoracic vertebrae, the mastoid process of the temporal bone, and the spinous processes of the cervical and thoracic vertebrae. Of the three columns, the longissimus muscle is the most extensive, whereas the spinalis muscle is the least developed. The primary function of the erector spinae is to maintain posture and support the body in an upright position.

### Spinal nerve

The spinal nerve is formed by the union of the anterior and posterior roots and remains attached to the lateral aspects of the spinal cord. Strictly speaking, “spinal nerve” specifically describes the short segment formed after the merging of the nerve roots, but before any branches are given off. There are twelve pairs of thoracic spinal nerves. At the thoracic level, spinal nerves exit the spinal canal through the intervertebral foramen, just below the pedicle of their corresponding vertebra. The spinal ganglion is a cluster of nerve cell bodies located in the posterior root and appears as its enlarged section. Spinal nerve branches include the anterior and posterior rami, the communicating branch, and the meningeal branch. At the thoracic level, the anterior ramus of the spinal nerve becomes the intercostal nerve. It first runs deep to the intercostal fascia, then courses between the internal and innermost intercostal muscles, and eventually supplies the thoracic wall and upper abdomen. The anterior cutaneous branch divides near the rib angle to give rise to the lateral cutaneous branch, which innervates the lateral chest wall, and also forms several muscular branches supplying the intercostal muscles. The posterior ramus of the spinal nerve divides into medial and lateral branches. The medial branch travels posteriorly, then turns medially along the border of the multifidus and passes between the multifidus and semispinalis muscles. In contrast, the lateral branch runs beneath the intertransverse ligament. Both medial and lateral cutaneous branches pierce the thoracic fascia. Additionally, the medial branches pass through the tendons of the back muscles^[13]^. The posterior branch of the spinal nerve is a key target for the effects of erector spinae plane block (ESPB). The meningeal branch arises from the anterior ramus and contains autonomic fibers from the same segment. The recurrent branch of each spinal nerve enters the spinal canal on the ventral aspect of the nerve within the intervertebral foramen and then divides into transverse, ascending, and descending branches. The communicating branch of the spinal nerve serves as an important connection between somatic spinal nerves and the sympathetic trunk, passing superior to the costotransverse ligament.

### Several important spaces in ESPB and PVB

**Figure 2.**
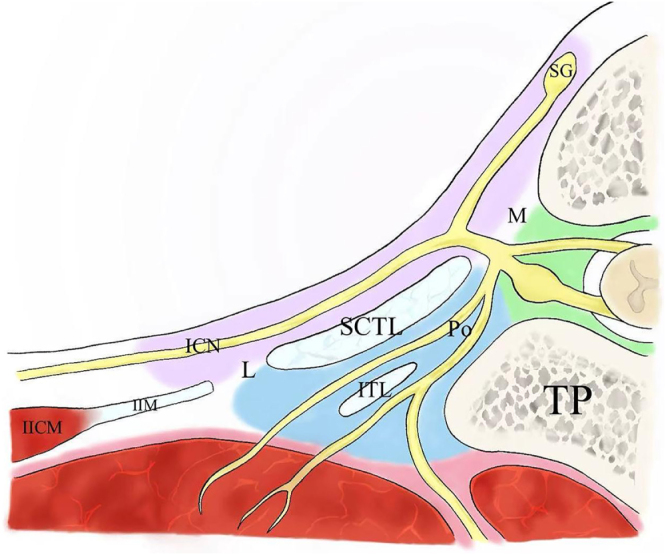
Local anatomy of the spine and its surrounding structures in cross-section. ICN: intercostal nerve (anterior branch of spinal nerve); IICM: internal intercostal muscle; ITL: transverse process ligament; L: lateral slit; M: medial slit; SG: sympathetic ganglia; TP: transverse process of vertebra; SCTL: superior costotransverse ligament; Po: posterior branch of spinal nerve; green areas are the intervertebral foramen and intervertebral space; red areas are erector spinae plane compartment; blue areas are the retro-SCTL space; pink areas are the paravertebral space.

The posterior space of the superior costotransverse ligament (SCTL) is situated between the SCTL, the transverse process of the vertebra, and the erector spinae muscle plane compartment. It communicates with the intervertebral foramen, intervertebral disc space, and paravertebral space (Fig. 2). The intervertebral foramen contains the spinal nerves and nerve roots^[14]^. The thoracic paravertebral (TPV) space lies on both sides of the thoracic spine. Its medial boundary is formed by the vertebral bodies, intervertebral discs, and intervertebral foramina, which connect to the epidural space. The TPV space gradually narrows as it extends into the intercostal space. This transition occurs near the costotransverse joint. Posteriorly, the TPV space is bounded by the transverse processes, ribs, and SCTL^[15]^. The TPV space is separated from adjacent segments by the ribs and transverse processes of each thoracic vertebra. Local anesthetics injected into these spaces spread and block the spinal nerves.

## Clinical effects of ESPB and PVB in thoracic and breast surgery

### ESPB

Numerous clinical trials have demonstrated that ESPB provides safe and effective analgesia following thoracic and breast surgery. It reduces the need for opioid analgesics, which may help lower the risk of opioid dependence^[9,10,16–29]^. Most clinical research on ESPB in this context has primarily focused on video-assisted thoracoscopic surgery (VATS) and breast surgery. Accordingly, this review examines the clinical effects of ESPB specifically in VATS and breast surgery (Table 1). Observational outcomes – including postoperative pain scores, opioid consumption, complication rates, and hospital length of stay – were analyzed to assess the clinical effectiveness of ESPB.

**Table 1:** Application of ESPB in thoracic surgery

Authors	Type of surgery	Analgesic methods and patient number	Assessment methods	Detection time	Results	Conclusions
Chun-Sung Sung 2024^[15]^	VATS	ESPB (N = 50), ICNB (N = 50)	VAS, morphine consumption	1, 24, 48 h	Postoperative VAS changes at 1, 24, and 48 h after surgery were also comparable between the two study groups. Both groups had low median scores (<4.0) at all time points (all *P* > 0.05). Patients in the ESPB group required statistically non-significant higher 48-h morphine consumption [3 (0–6) vs. 0 (0–6) mg in the ESPB group and ICNB group, respectively; *P* = 0.135] and lower numbers of oral rescue analgesic (0.4 ± 1.2 vs. 1.0 ± 1.8 in the ESPB group and ICNB group, respectively; *P* = 0.059).	Both anesthesiologist-administered ultrasound-guided ESPB and surgeon-administered VATS ICNB were effective analgesic techniques for patients undergoing VATS for tumor resection.
Wang Hejie 2019^[10]^	Radical mastectomy	SAPB group (N = 50), ESPB group (N = 50), control group (N = 50)	VAS, the intraoperative dosages of propofol and remifentanil, press times and sufentanil cumulative dosage of PCIA in 48 h after operation.	2,4,8,12,24,48 h	In all the three groups, the VAS scores at rest and coughing increased first and then decreased 2–48 h after operation. The VAS scores in SAPB group and ESPB group were lower than that in control group (*P* < 0.05), whereas, no significant difference was observed between SAPB group and ESPB group (*P* > 0.05).	Both SAPB and ESPB can provide good and safe analgesia for radical mastectomy, with equivalent performances in analgesia and adverse effect.
Nan Chen 2020^[18]^	VATS	PVB (N = 25), ESPB (N = 25), ICNB (N = 25)	VAS, morphine consumption	0, 2, 4, 8, 24, 48 h	PVB group had significantly lower VAS scores at rest and while coughing than ESPB group at 0, 2, 4, 8 h postoperatively and than ICNB group at 8 h postoperatively.	Ultrasound-guided multiple-injection PVB provided superior analgesia to ICNB and single-injection ESPB, while ICNB and single-injection ESPB were equally effective in reducing pain after thoracoscopic surgery.
Hong Zhao 2020^[19]^	VATS	ESPB (N = 33), PVB (N = 33)	Postoperative oxycodone consumption at 48 h. Resting pain score. QoR	48 h	Intraoperative use of sufentanil and remifentanil were comparable between these two groups. Pain scores, oxycodone rescue and Quality of Recovery (QoR) 15 on postoperative day 1 and 2 were equivalent between these two groups.	Ultrasound-guided ESPB applied before video assisted thoracic surgery was non-inferior in analgesic effect compared with PVB in terms of pain score, analgesic rescue consumption and quality of recovery.
Bahadir Ciftci 2019^[20]^	VATS	ESPB group (N = 30) and a control group (N = 30)	VAS, morphine consumption	0, 2, 4, 8, 16, 24 h	The opioid consumption at 1, 2, 4, 8, 16, and 24 h and the active and passive VAS scores at 0, 2, 4, 8, 16, and 24 h were statistically lower in the ESPB group at all of the time periods when compared with the control group (*P* < 0.05).	A preemptive single-shot ESPB may provide effective analgesia management after VATS.
Ozlem Turhan MD 2020^[21]^	Thoracoscopic surgery	EPSB (N = 35), TPVB (N = 35), ICNB (N = 35)	VAS, morphine consumption	1, 12, 24 h	Dynamic pain scores were significantly lower in GTPV compared to GESP and GICN for 24 h (*P* <0.017). Dynamic pain scores in GICN were significantly lower for 12 h compared to GESP (*P* < 0.017). Morphine consumption for the first 24 h was similar in GICN and GTPV, while it was significantly lower in GICN and GTPV in comparison to GESP (*P* < 0.017). Rescue analgesic requirement and side effects were similar between groups.	All three blocks can obtain sufficient analgesia after VATS, however TPVB appears to be the preferable method compared to ESPB and ICNB with a more successful analgesia and with less morphine consumption
A. Eskandr 2022^[56]^	Cancer breast surgeries	TPVB (N = 20), PECS (N = 20), ESPB (N = 20), and control (N = 20)	The duration of analgesia	48 h	ESPB has a shorter duration of analgesia than PECS block with no significant statistical difference compared with group TPVB. Morphine consumption is increased in ESPB compared to the PECS group, with an insignificant difference compared to group TPVB. There was an insignificant difference between the groups concerning hemodynamics and complications, with one pneumothorax case reported in the TPVB group.	PECS and ESPB represent a good alternative to TPVB for post-mastectomy analgesia with a superior analgesic effect of PECS block regarding opioid consumption, duration of the analgesia, and VAS score.
AntoinePremachandra 2022^[9]^	Breast cancer surgery	EPSB (N = 92), TPVB (N = 92)	Morphine consumption in the PACU, VAS	24 h	The proportion of morphine titration in the PACU was higher in the ESPB group than in the TPVB group	ESPB is less effective in preventing morphine consumption in the PACU than TPVB. Our findings do not support the use of ESPB as the first-line regional an aesthesia for major breast cancer surgery. Randomized trials comparing ESPB and TPVB are needed.
Ighor Pallu 2022^[22]^	VATS	ESPB (N = 42), LBA (N = 50)	NVS, opioid consumption	6, 12, 24 h	Mean opioid consumption in the BAL and ESPB groups in the POI was 12.9 (± 10.4) mg vs 14.9 (±10.2) mg, respectively, with *P* = 0.416.	Local anesthetic block and ESP block techniques showed similar results in terms of low pain scores and opioid consumption during the period evaluated.
Sevim Cesur 2023^[27]^	Radical mastectomy surgery	PECS (N = 32), ESPB (N = 35)	NRS, opioid consumption	6, 12, 24 h	Postoperative total morphine consumption in the first 24 h was significantly higher in the PECS group (*P* < 0.001). The ESPB group exhibited significantly reduced morphine consumption at all postoperative time points. Numeric rating scale scores were lower in the ESPB group at 6, 12, and 24 h postoperatively at rest and when coughing.	Ultrasound-guided bi-level ESPBs provided better postoperative analgesia than PECS blocks after radical mastectomy surgery.
Matthew W Swisher 2020^[16]^	Breast surgery	PVB (N = 50), EPSB (N = 50)	NRS, opioid consumption	24 h	Both pain scores and opioid consumption were higher in subjects with ESPBs than PVBs	Without a dramatic improvement in safety profile for ESPBs, it appears that PVBs are superior to ESPBs for postoperative analgesia after non-mastectomy breast surgery
Caner Genc 2022^[28]^	Breast cancer surgery	ESPB (N = 30), PSPB (N = 30), control (N = 30)	Opioid consumption, VAS	24 h,3 mon	Postoperative 24-h morphine consumption, visual analog scale scores at rest and at abduction, and intraoperative remifentanil consumption were lower in the ESPB and PSPB groups than in the control group.	US-guided ESPB and PSPB performed in patients who underwent breast cancer surgery showed similar and modest analgesic effects on the postoperative opioid consumption and acute and chronic pain scores.
Mu¨ rsel Ekinci 2020^[23]^	VATS	ESPB (N = 30), SAPB (N = 30)	Opioid consumption, VAS	0–8, 8–16, 16–24 h	Intraoperative and postoperative opioid consumption at 0–8, 8–16, and 16–24 h and rescue analgesic use were significantly lower in the ESPB group (*P* < 0.05). Static/dynamic VAS scores were significantly lower in the ESPB group (*P* < 0.05).	US-guided ESPB may provide better pain control than SAPB after VATS. Question.
Demet Lafli Tunay 2023^[29]^	Breast reduction surgery	ESPB (N = 30), control (N = 30)	24-h total morphine consumption	24 h	The 24-h total morphine consumption was significantly lower in the ESPB group vs. the sham group (mean ± SD, 6.7 ± 3.9, and 13.9 ± 5.7 mg, respectively; *P* < 0.001). Compared with sham block, ESPB reduced pain scores, intraoperative opioid consumption, supplement analgesic requirements, delayed time to first PCA request, and improved functional recovery and patient satisfaction.	In breast reduction surgery, preoperative single-shot ESPB reduces perioperative opioid consumption and provides adequate pain relief within 24 h postoperatively compared to systemic analgesics alone.
Ahmed M. Elewa 2022^[55]^	Breast surgery	ESPB, PVB, general anesthesia	Opioid consumption, VAS	24 h	The ESPB (4.9 ± 1.2 mg) and PVB (5.8 ± 1.3 mg) groups had significantly lower total morphine consumption than the control group had (16.4 ± 3.1 mg; *P* < 0.001). The postoperative visual analog scale scores were lower in the ESPB and PVB groups than in the control group on the first 24 h after the procedure (*P* < 0.001).	ESPB and PVB provide effective postoperative analgesia for women undergoing MRM. The ESPB appears to be as effective as the PVB.
Lingling Sun 2022^[24]^	VATS	ICNB (N = 59), TPVB + ICNB (N = 56), or ESPB + ICNB (N = 59)	Opioid consumption, VAS	2, 6, 8, 12, 24, 48 h	The VAS when coughing in Group T were lower than that in Group C (mean difference = 0.15, 95%CI, 0.02–0.29; *P* = 0.028). While no difference was found when comparing Group E with Group C or Group T (*P* > 0.05). There was no difference between the three groups in the sufentanil consumption (within 24 h, *P* = 0.472; within 48 h, *P* = 0.158) and supplementary analgesic requirements (*P* = 0.910).	The present randomized trial showed that the analgesic effect of TPVB + ICNB was superior to that of INCB after VATS, the analgesic effect of ESPB was equivalent to that of TPVB and ICNB.
Ahmed Mohamed Mohamed Rabah Abdella 2022^[31]^	Breast cancer surgeries	Standard volume ESPB, high volume ESPB, GA only group	VAS	12 h	VAS at rest 12 h after surgery was less in both intervention groups compared to the control (1.75 ± 0.79 vs. 1.6 ± 0.88 vs. 3.4 ± 1.96, *P* = 0.001). The LA had extended further in the high volume group than the standard volume group (11.20 ± 3.07 vs. 9.15 ± 2.54 vertebral levels; *P* = 0.027).	Preoperative ESPB is an excellent analgesic modality and it can also attenuate both postoperative agitation and sedation. Doubling the injectate volume enhances the craniocaudal spreading and may be useful for surgeries requiring multiple dermatomes.
Jie Zhang 2023^[25]^	Video-assisted thoracoscopic lobectomy	General anesthesia group (group A, n = 46) and ESPB combined with general anesthesia group (group B, n = 48)	NRS	2, 6, 12, 24, 48 h	Compared with group A, rest and cough NRS score at 2, 6, 12, 24, and 48 h after surgery, frequency of PCIA use at 24 h after surgery, frequency of rescue analgesia were significantly decreased in group B (*P* < 0.05). There was no significant difference in NRS scores of rest and cough at PACU after operation between 2 groups after surgery at post anesthesia care unit (*P* > 0.05).	Ultrasound-guided erector spinae plane block can significantly reduce acute post-surgical pain, can significantly reduce the severity of chronic pain in patients underwent video-assisted thoracoscopic lobectomy
Wei Wu 2023^[26]^	Uniportal thoracoscopic lobectomy	ESPB (N = 44), SAPB (N = 46), control (N = 47)	The consumption of sufentanil, NRS	24 h	No significant difference was observed in the consumption of sufentanil during the first 24 h following surgery between the ESPB and SAPB groups. There were no significant differences in AUC of NRS scores during rest and movement between the ESPB and SAPB group.	Although the differences between the two groups are not statistically significant, both the ESPB and SAPB demonstrate effective reduction in postoperative opioid consumption and the need for rescue analgesics compared to the control group.

#### ESPB for VATS

There is ongoing debate regarding the clinical effectiveness of erector spinae plane block (ESPB) in video-assisted thoracoscopic surgery (VATS). Several studies have reported that paravertebral block (PVB) is more effective than ESPB for pain reduction after VATS^[19,22,25]^, whereas Zhao *et al*^[20]^ found no significant difference in analgesic efficacy between the two techniques. In addition to ESPB and PVB, other regional analgesic techniques such as intercostal nerve block (ICNB), serratus anterior plane block (SAPB), and local anesthesia are also commonly used for postoperative pain control in VATS. Both ICNB and single-shot ESPB have been shown to effectively reduce pain after thoracoscopic surgery^[19]^. Compared with SAPB, single-shot ESPB is associated with lower visual analogue scale (VAS) scores and reduced opioid consumption^[10,24]^. Local anesthesia and ESPB produce similar outcomes in terms of postoperative pain scores and opioid consumption^[23]^. Furthermore, PVB is associated with a higher risk of complications, such as hematoma and pneumothorax, compared with ESPB^[9,13,19]^. Thus, ESPB is considered safer^[19,25]^ and is associated with fewer complications^[21]^.

#### ESPB for breast surgery

ESPB is an effective analgesic technique that reduces perioperative opioid use and provides adequate pain relief within 24 hours after surgery^[9,16,17,27–29,31,32]^. However, the clinical efficacy of ESPB in breast surgery remains controversial. Wang *et al*^[9]^ reported that PECS block is more effective than ESPB for pain control after breast surgery, whereas Cesur *et al*^[28]^ found ESPB to be superior. Other common regional anesthesia techniques for breast surgery include SAPB, TPVB, and PSPB. Both SAPB and ESPB provide effective and safe analgesia for patients undergoing radical breast cancer surgery, with similar efficacy and rates of adverse events^[10]^. ESPB is less effective than TPVB in reducing morphine consumption in the post-anesthesia care unit (PACU), and is therefore not recommended as a first-line regional anesthesia technique for extensive breast cancer surgeries^[27]^. However, some studies suggest that both ESPB and PVB provide effective postoperative analgesia for women undergoing modified radical mastectomy (MRM), and that ESPB may be as effective as PVB^[16]^. Ultrasound-guided ESPB and PSPB demonstrate similar, moderate analgesic effects on postoperative opioid consumption as well as acute and chronic pain scores in patients undergoing breast cancer surgery^[29]^.

### PVB

PVB is a widely used technique for postoperative analgesia following thoracic surgery. Numerous clinical trials have demonstrated that PVB provides safe and effective analgesia after thoracic and breast surgery (Table 2). The main indications for PVB in this context are VATS and breast surgery. Observational outcomes such as postoperative pain scores, opioid consumption, and complication rates were statistically analyzed to assess the clinical efficacy of PVB.

**Table 2:** Application of PVB in thoracic surgery

Authors	Type of surgery	Analgesic methods and patient number	Assessment methods	Detection time	Results	Conclusions
Yan Wang 2022^[35]^	VATS	SAPB (N = 49), PVB (N = 50)	VAS	24,48 h	No significant differences in VAS scores at rest and cough at first 48 h, 3 months, and 6 months postoperatively between the SAPB group and PVB group were detected (all *P* > 0.05).	The ultrasound-guided SAPB was not inferior to PVB in alleviating postoperative pain following the VATS with fewer complications and higher patient satisfaction.
Nan Chen 2020^[18]^	Thoracoscopic surgery	PVB (N = 25), ESPB (N = 25), ICNB (N = 25)	VAS, morphine consumption	0, 2, 4, 8, 24, 48 h	PVB group had significantly lower VAS scores at rest and while coughing than ESPB group at 0, 2, 4, 8 h postoperatively and than ICNB group at 8 h postoperatively.	Ultrasound-guided multiple-injection PVB provided superior analgesia to ICNB and single-injection ESPB, while ICNB and single-injection ESPB were equally effective in reducing pain after thoracoscopic surgery.
Hong Zhao 2020^[19]^	VATS	ESPB (N = 33), PVB (N = 33)	Postoperative oxycodone consumption at 48 h, resting pain score, QoR	24 h	There was no difference between NRS scores and opioid consumption at any hour between the groups.	While ESPB and QLB-II are not significantly different, they improve analgesia quality in patients undergoing LC.
Wengang Ding 2018^[54]^	Thoracoscopic lobectomy	PVB-R (N = 38), PVB-RD (N = 38), TEA (N = 38)	The dose and first time of postoperative analgesia, verbal rating score (VRS)	48 h	Compared to the PVB-R group, the dose of postoperative analgesia and VRS were lower and the first time of postoperative analgesia were longer in the PVB-RD and TEA group. Patients in the PVB-RD group had a lower incidence of side effects compared to those in the TEA group.	Single-dose 0.5% ropivacaine combined with dexmedetomidine (1 ΰg/kg) PVB provides satisfactory postoperative pain control after thoracoscopic lobectomy, and can reduce the incidence of postoperative side effects.
Yavuz Gürkan 2019^[37]^	Breast surgery	ESPB (N = 25), PVB (N = 25), control (N = 25)	NRS, opioid consumption	1, 6, 12, 24 h	There was a statistically significant difference between ESP and Control groups (*P* < 0.001) and between PVB and Control groups (*P* < 0.001), while there was no difference between ESP and PVB groups (*P* > 0.05) for 24-hour morphine consumptions. There was a significant difference between PVB and Control groups for NRS at postoperative 1st and 6th hour (*P* = 0.018 and *P* = 0.027, respectively).	This study has shown that US guided ESP block and PVB provided adequate analgesia in patients undergoing breast surgery and have an opioid sparing effect by reducing morphine consumption.
Zixiang Wu 2023^[36]^	VATS	PVB (N = 88), TEA (N = 88)	VAS	48 h	No significant difference in the visual analogue scale score was found between the 2 groups at rest (*P* = 0.395) or with coughing (*P* = 0.157). Additionally, there was no significant difference in the average pain score between these 2 states (*P* = 0.221).	PVB can provide pain relief that is similar to that of TEA but with no additional puncture pain, a shorter catheter placement time, and fewer side effects in patients undergoing video-assisted thoracoscopic surgery.
Aneurin Moorthy 2022^[32]^	VATS	ESPB (N = 40), PVB (N = 40)	(QoR-15) at 24 h	24 h	Median (25e75%) QoR-15 at 24 h was higher in ESP (n¼37) compared with PVB (n¼37): 118 (106e134) vs 110 (89e121) (P¼0.03) and at 48 h: 131 (121e139) vs 120 (111e133) (P¼0.03).	Compared with video-assisted, surgeon-placed paravertebral catheter, erector spinae catheter improved overall QoR-15 scores at 24 and 48 h but without differences in pain or opioid consumption after minimally invasive thoracic surgery.
Matthew WSwisher 2020^[16]^	Breast surgery	PVB (N = 50), EPSB (N = 50)	NRS, opioid consumption	24 h	Both pain scores and opioid consumption were higher in subjects with ESPBs than PVBs	Without a dramatic improvement in safety profile for ESPBs, it appears that PVBs are superior to ESPBs for postoperative analgesia after non-mastectomy breast surgery
Shun Wang 2023^[38]^	Breast surgery	PVB (N = 50), SAPB (N = 50), SA-TTMPB (N = 50)	QoR-15, NRS	24 h,48 h,72 h,7d	The QoR-15 total score of S group at 24 h, 48 h, 72 h and 7 days post-surgery was significantly lower in groups P and ST, while there was no significant difference between groups P and ST.	SAPB combined with transverse thoracic muscle plane block and PVB both have better effects than serratus anterior plane block alone in improving patients’ early post-operative recovery quality, and also have an advantage in improving early post-operative pain.
Vishal Uppal 2017^[56]^	Unilateral mastectomy	Single (N = 32) or multiple (N = 38) injections of a PVB	The median (interquartile range) dermatomal spread	20 min	The median (interquartile range) dermatomal spread was not significantly different for the single-injection group (5^[4-6]^) compared with the multiple-injection group	An ultrasound-guided single-injection PVB provides equivalent dermatomal spread and duration of analgesia compared with a multiple-injection PVB. The single-injection technique takes less time to perform and hence may be preferred over a multiple-injection technique.
Ahmed M.Elewa 2022^[55]^	Breast surgery	ESPB, PVB, general anesthesia	Opioid consumption, VAS	24 h	The ESPB (4.9 ± 1.2 mg) and PVB (5.8 ± 1.3 mg) groups had significantly lower total morphine consumption than the control group had (16.4 ± 3.1 mg; *P* < 0.001).The postoperative visual analog scale scores were lower in the ESPB and PVB groups than in the control group on the first 24 h after the procedure (*P* < 0.001).	ESPB and PVB provide effective postoperative analgesia for women undergoing MRM. The ESPB appears to be as effective as the PVB.
Lingling Sun 2022^[24]^	VATS	CNB (N = 59), TPVB + ICNB(N = 56) or ESPB + ICNB (N = 59)	Opioid consumption, VAS	2, 6, 8, 12, 24, 48 h	The VAS when coughing in Group T were lower than that in Group C (mean difference = 0.15, 95%CI, 0.02–0.29; *P* = 0.028). While no difference was found when comparing Group E with Group C or Group T (*P* > 0.05). There was no difference between the three groups in the sufentanil consumption(within 24 h, *P* = 0.472; within 48 h, *P* = 0.158) and supplementary analgesic requirements (*P* = 0.910).	The present randomized trial showed that the analgesic effect of TPVB + ICNB was superior to that of INCB after VATS, the analgesic effect of ESPB was equivalent to that of TPVB and ICNB.

#### PVB for VATS

The clinical effects of ESPB and PVB in VATS surgery have been discussed previously. Additionally, epidural catheters can improve outcomes at 24 and 48 hours after minimally invasive thoracic surgery compared with paravertebral catheters placed under surgical video guidance; however, overall Quality of Recovery-15 (QoR-15) scores were similar, with no significant differences in pain or opioid consumption^[35]^. Other commonly used analgesic techniques for VATS include intercostal nerve block (ICNB), serratus anterior plane block (SAPB), and local anesthesia. Ultrasound-guided SAPB provides postoperative analgesia comparable to PVB after VATS, but with fewer complications^[36]^. PVB provides pain relief similar to that of thoracic epidural analgesia (TEA) for patients undergoing VATS, without the additional puncture pain and with fewer side effects^[37]^.

#### PVB for breast surgery

Multiple studies have demonstrated that PVB provides effective analgesia after breast surgery^[38]^. The comparative effects of ESPB and PVB on postoperative analgesia in breast surgery remain controversial, as previously discussed. Wang *et al*^[39]^ reported that PVB is more effective than SAPB in improving early postoperative recovery and alleviating early postoperative pain.

## Mechanism and diffusion range of ESPB and PVB

### ESPB

ESPB achieves analgesia by injecting local anesthetics to block the corresponding nerves, utilizing their pharmacological properties. Theoretically, due to its unique anatomical features, local anesthetics injected via ESPB can diffuse in five directions: into the paravertebral space through the connective tissue between transverse processes; into the epidural space via the intervertebral foramen; cranio-caudally to adjacent vertebrae; posteriorly to block the dorsal branch of the spinal nerve in the erector spinae muscle; and laterally into the intercostal space^[8]^. Imaging studies in living subjects often demonstrate that ESPB injectate spreads to the dorsal branch of the spinal nerve, paravertebral space, intervertebral foramen, and epidural cavity^[40,41]^. Nearly all anatomical studies have shown that ESPB injections can spread posteriorly to the dorsal branch of the spinal nerve^[14,33,42–46]^. In theory, local anesthetics may block the spinal nerve roots, the anterior (intercostal nerve) and posterior branches, communicating branches, and meningeal branches to varying degrees. However, the actual spread and mechanism of action remain controversial according to existing literature. Some researchers, including Bonvicini^[42]^ and Diwan, argue that ESPB injectate can spread to the paravertebral space, while others, such as Harbell^[43]^, DeLara González^[44]^, Lim^[45]^, Aponte^[33]^, and Yang^[34]^, believe that this spread is rare or unlikely. Similarly, Bonvicini^[42]^ and Sanjib Das Adhikary^[30]^ report that ESPB injectate can reach the anterior branch of the spinal nerve, in contrast to Harbell^[43]^, Lim^[45]^, and Aponte^[33]^, who assert that such spread is very rare or does not occur. There is currently a lack of anatomical studies regarding whether ESPB can block the spinal nerve roots, communicating branches, and meningeal branches. In addition to anatomical mechanisms, the volume of local anesthetic is considered an important factor influencing the extent of ESPB diffusion^[47,48]^. A 20 mL injection is generally considered safe and effective for nerve blocks and is widely used in both clinical and anatomical studies. Increasing the injectate volume up to 30 mL appears to expand the spread within back muscles and fascial layers more than in the paravertebral space^[47]^.

### PVB

TPVB achieves blockade of the spinal nerve roots by injecting local anesthetics into the lateral aspect of the thoracic foramen^[8]^. Local anesthetics can then reach the paravertebral or epidural space via the intervertebral foramen. Paravertebral block directly targets the paravertebral space adjacent to the neuraxis, and its analgesic effects are comparable to those of epidural block^[28]^. PVB provides effective analgesia for both somatic and visceral pain. The anterior histological diffusion pattern of ESPB injectate is similar to that of paravertebral block^[42]^. In the absence of detailed anatomical studies on PVB, the anterior diffusion mechanism observed with ESPB provides valuable reference for understanding PVB mechanisms and highlights the need for further anatomical research.

## Discussion

Although numerous clinical trials suggest that ESPB and PVB are safe and effective for thoracic and breast surgery, particularly in VATS and breast procedures^[9,10,16–29,31,32]^, a critical appraisal of the evidence base reveals several weaknesses that limit the generalizability of these findings. Many included randomized controlled trials featured relatively small sample sizes, inconsistent randomization and blinding protocols, and marked heterogeneity in patient selection, analgesic regimens, and outcome measurements. Moreover, important confounders, such as operator experience and use of adjunct analgesic interventions, are often not adequately controlled for, and follow-up periods are generally short. These methodological shortcomings limit the validity and clinical applicability of the reported reductions in opioid use and complication rates. The lower incidence of complications such as hematoma and pneumothorax with ESPB compared to PVB, for example, may be subject to reporting and detection bias, particularly in smaller cohorts. To establish the true benefit and risk profile of these two techniques, future research should prioritize large-scale, rigorously designed multicenter RCTs with standardized interventions and outcome metrics.

The literature on the anatomical mechanisms of ESPB and PVB also suffers from important limitations that impact the translation of anatomical findings into clinical practice. Existing studies are dominated by cadaveric dye injections and small-sample imaging studies, which are highly variable in technique and reporting. The diffusion patterns observed in cadaveric studies using dye or contrast agents may not accurately reflect living tissue characteristics, and tissue deformation in cadavers may further alter spread. Some anatomical studies support the hypothesis that ESPB can reach the paravertebral space and thus provide both anterior and posterior ramus blockade, but results are inconsistent and sometimes contradictory. Critically, there is a lack of high-quality evidence directly linking these anatomical observations to clinically meaningful analgesic effects or complication profiles. Accordingly, future research should focus on (1) standardizing injection techniques and volumes in anatomical models, (2) employing multimodal imaging in live subjects to validate diffusion patterns, and (3) establishing robust correlations between anatomical spread and clinical endpoints such as analgesic efficacy and adverse event rates.

Erector spinae plane block (ESPB) exemplifies the complex interplay between local anesthetic dosing and tissue anatomy. There is growing evidence that total dose (the product of concentration and volume) is the primary determinant of both effectiveness and safety, with a clear dose–response relationship^[49,50]^. While a 20 ml volume is widely used and generally regarded as safe and effective^[49,51]^, both clinical and anatomical studies emphasize the importance of calibrating total dose and administration method to patient anatomy and procedural needs, in order to maximize analgesic benefit and minimize systemic toxicity^[50,52]^. Excessive volume or concentration can increase both the extent and the adverse effect profile of the block^[50,52]^. Accordingly, an individualized dosing strategy – tailored to patient characteristics and clinical goals – is recommended, and further prospective trials are needed to optimize dosing guidelines and clarify the dose–response relationship^[49,50]^.

Beyond dosing, patient-specific physiology and the physicochemical properties of local anesthetics also play an important role in the outcomes of ESPB. Agents such as bupivacaine and ropivacaine act by reversibly blocking voltage-gated sodium channels, with their clinical performance – such as onset, duration, tissue penetration, and systemic absorption – being influenced by characteristics like lipid solubility, pKa, and protein binding^[53]^. Importantly, local anesthetics may be temporarily sequestered within anatomical planes that may be rich in connective or adipose tissue, potentially delaying systemic absorption and partially accounting for interindividual variability in clinical response^[54]^. However, the hypothesis that tissue characteristics such as fat content impact local anesthetic dynamics currently remains speculative and is supported by limited evidence. Further studies are required to clarify these relationships. A deeper understanding of both pharmacology and local anatomy will be critical for refining ESPB techniques and achieving optimal, patient-tailored analgesia^[53,54]^.

To address the current gaps and limitations, future research priorities are categorized as follows:

Core research priorities (necessary): (1) Conduct large-scale, multicenter randomized controlled trials (RCTs) with rigorous methodology and standardized interventions to clarify the clinical benefits and risks of ESPB and PVB. (2) Use multimodal imaging in live subjects to validate injectate diffusion patterns and establish robust correlations between anatomical spread and meaningful clinical outcomes, such as analgesic efficacy and adverse event rates.

Ancillary research areas (supportive): (1) Standardize injection techniques and volumes in anatomical or cadaveric models to improve comparability across studies. (2) Undertake prospective trials to optimize dosing guidelines and clarify the dose–response relationship for ESPB and PVB. (3) Further investigate how patient-specific tissue characteristics and pharmacological properties of local anesthetics influence distribution and clinical effectiveness, to advance personalized analgesic strategies.

## Conclusion

The current evidence on ESPB and PVB for post-thoracic analgesia reveals a fundamental paradox: while PVB often demonstrates superior analgesic efficacy, it is associated with higher risks of complications, whereas ESPB, despite an improved safety profile, may provide relatively less consistent analgesia. These differences can be mechanistically explained on three levels. First, anatomical factors are paramount: PVB delivers local anesthetic in close proximity to the spinal nerves and pleura within the paravertebral space, ensuring reliable nerve blockade but increasing the risk of pleural or vascular injury. ESPB, by contrast, is performed in a more superficial fascial plane, further from critical neurovascular structures, which enhances safety but makes effective anterior spread of local anesthetic – and thus robust nerve blockade – more dependent on individual variations in fascial integrity and connectivity. Second, the spread characteristics of local anesthetic are closely tied to fascial continuity and resistance; while PVB benefits from the relatively confined paravertebral anatomy that supports localized and predictable drug spread, ESPB outcomes vary substantially due to patient-specific anatomy, fascial plane variability, and even differences between spinal segments. Third, significant methodological heterogeneity and operator-dependent technical factors across published studies further amplify these clinical differences: variation in block technique, dosing, equipment, patient selection, and the learning curve for anatomical identification all directly influence the efficacy and safety of both techniques. Collectively, these anatomical, methodological, and technical factors underlie the observed trade-off between PVB’s more potent but riskier analgesia and ESPB’s greater safety but less predictable effect.

To move the field forward, future research should prioritize: (1) standardized live imaging studies to directly track injectate spread in both ESPB and PVB; (2) large, multicenter prospective trials comparing different drug concentrations, volumes, and needle approaches under controlled conditions; (3) validated assessment tools to quantify inter-operator variability and procedural learning curves; and (4) anatomical and histological investigations of the relevant fascial layers – including fascial thickness, collagen fiber orientation, and connective tissue density – with particular emphasis on the relationship between fascial plane variations and the course of posterior spinal nerve branches, to determine how these microstructural characteristics and anatomical relationships influence local anesthetic spread and block efficacy.

Building on these findings, our review offers several novel contributions to the existing literature:

First, by systematically integrating both clinical and anatomical studies, we provide a unique and up-to-date synthesis of ESPB and PVB in thoracic and breast surgery, clarifying previous ambiguities regarding their relative efficacy, anatomic mechanisms, and safety profiles. Our review also explicitly addresses outstanding controversies regarding the variability of local anesthetic spread and its implications for clinical effect and complication risk, a dimension less systematically explored in prior reviews.

Second, we highlight that while PVB may offer superior and more predictable analgesic efficacy due to its proximity to the paravertebral space, ESPB’s more superficial approach results in a more favorable safety profile, with lower rates of serious complications such as pneumothorax or hematoma. We also emphasize that the multidirectional and variable spread of local anesthetic with ESPB, largely dependent on individual anatomy and fascial plane integrity, explains the observed trade-off between efficacy and safety between these two techniques.

Third, our review prioritizes research directions for the field: (1) the need for large-scale, well-designed multicenter randomized controlled trials with standardized methodologies, and (2) the importance of using multimodal imaging in live subjects to validate the anatomical pathways of injectate and directly link anatomical patterns to meaningful clinical endpoints such as analgesic success and adverse events.

The following learning points can be drawn for clinical practice and research:

1. PVB generally provides stronger and more predictable analgesia but at the cost of a higher complication rate; ESPB, though variable in efficacy, offers improved safety and is especially valuable in higher-risk or technically challenging patients.
2. The direction and consistency of local anesthetic spread is a key determinant of block success; multidirectional spread with ESPB explains its less predictable efficacy.
3. Clinical decision-making should be individualized, balancing the risk profile, patient anatomy, and procedural needs.
4. Standardization in block technique, training, and reporting is critical for improving outcomes and advancing the field.
5. A major research priority is establishing robust links between anatomical findings and clinical outcomes through advanced imaging and high-quality trials.

Several limitations of this review should be acknowledged. As a narrative rather than a systematic review, the selection of literature was inherently subjective and may have introduced selection bias. Study quality and risk of bias were not formally assessed, and no quantitative synthesis was performed. These limitations should be considered when interpreting the present conclusions.

## Data Availability

Not applicable.
